# A multimodal experimental dataset on agile software development team interactions

**DOI:** 10.1016/j.dib.2025.111828

**Published:** 2025-06-24

**Authors:** Diego Miranda, Carlos Escobedo, Dayana Palma, Rene Noel, Adrián Fernández, Cristian Cechinel, Jaime Godoy, Roberto Munoz

**Affiliations:** a Escuela de Ingeniería Informática, Unviersidad de Valparaíso, Valparaíso, Chile; b Centro de Ciências, Tecnologias e Saúde, Universidade Federal de Santa Catarina, Araranguá, Brazil

**Keywords:** Multimodal analytics, Agile development, Collaborative learning, User stories, Team dynamics, Behavioural synchrony

## Abstract

Studying collaborative dynamics in agile development teams requires multi- modal data that captures verbal and non-verbal communication. However, few experimental datasets provide this level of depth in real or simulated teamwork contexts. This article presents a multimodal dataset with experimental data collected during controlled sessions involving simulated agile development teams, each composed of four computer science students. A total of 19 groups (76 different participants) were organized, each participating in two collaborative activities: one without a coordination technique and another using the Planning Poker method. Three of these teams were designated as control groups. The resulting dataset includes audio recordings of verbal interactions and non- verbal behaviour data, such as body posture, facial expressions, visual attention, and gestures, captured using MediaPipe, YOLOv8, and DeepSort. It also contains time-aligned automatic transcriptions generated with WhisperX, attention logs, mimicry labels, and surveys on perceived equity in interactions. This re- source aims to provide a comprehensive view of collaborative behaviour in agile contexts, supporting both qualitative analysis of interactions and the development of predictive models of group performance. The dataset explores how shared visual attention and behavioural synchrony influence team effectiveness and decision-making through this multimodal approach. This work contributes a unique dataset valuable to researchers across multiple fields of study.

Specifications Table

**Table:** 

Subject	Computer Sciences
Specific subject area	Team dynamics and agile methodologies.
Type of data	Audio recordings of sessions (.wav) Processed summary videos (.mp4) Participant tracking data (.userTracker.parquet) Face tracking data (.faceTracker.parquet) Body posture tracking data (.poseTracker.parquet) Hand gesture tracking data (.handsTracker.parquet) Head orientation/gaze direction data (.faceDirection.parquet) Visual attention data (.ObservedUser.parquet) Time-aligned automatic transcriptions (.transcription.parquet) Mimicry annotations (.xlsx) Perceived equity surveys (.xlsx) Comparative analysis results (CSV/PNG files in .zip).
Data collection	Audiovisual recordings and survey.
Data source location	Universidad de Valparaíso, Chile
Data accessibility	Repository name:DataSet Fondecyt1211905: • URL: https://data.mendeley.com/datasets/yw4my59xrg/ • DOI: 10.17632/yw4my59xrg.3 DataSet_Videos Fondecyt1211905: • URL: https://data.mendeley.com/datasets/rbpvjdpkby/ • DOI: 10.17632/rbpvjdpkby.1
Related research article	D. Miranda, R. Noel, J. Godoy, C. Escobedo, C. Cechinel, R. Munoz. Quantitative analysis of communication dynamics in agile software teams through multimodal analytics. Scientific Reports. 2025;15(1). https://doi.org/10.1038/s41598-025-91328-x

## 1 Value of the Data


 

• This dataset provides a solid foundation for studying collaborative dynamics in agile teams by leveraging multimodal data to capture verbal and non-verbal interactions. Verbal interactions include conversations during work sessions and automatically generated transcriptions of discussions, while non-verbal interactions encompass posture, facial expressions, and both facial and bodily mimicry.
• The dataset contains data collected through various methods: automatic analysis of audio using WhisperX^1^, and video analysis utilizing MediaPipe^2^, YOLOv8^3^, and DeepSort^4^. Additionally, it includes manual an- notations based on participant mimicry and perceptions gathered during the experimental activities. To ensure consistency across all tools, each data source was manually verified through a two-phase review process: first by a junior judge who conducted the initial verification, followed by a senior judge who adjusted and refined the details. Furthermore, to ensure consistency across tools, participant labelling was checked and corrected to guarantee that the audio and video data linked to each participant share the same identifier. This thorough verification procedure ensured that audio and video data associated with a given participant consistently share the same identifier throughout the dataset.
• It will enable the evaluation of whether collaboration patterns modelled from perceptions or game-based environments, as in [1] and [2], correspond to observable behaviours in multimodal data.
• The data captured using advanced tools such as WhisperX, YOLOv8, and MediaPipe enable the analysis of detailed interaction metrics. In addition, a visual verification process was conducted to eliminate potential errors, which is essential to ensure the highest possible data accuracy.
• This dataset can be used to train models for predicting the performance of collaborative teams in both academic and professional settings, as dis- cussed in group performance prediction studies [3].
• There are existing datasets that measure specific aspects of agile teams, such as task metrics, estimations, and completion times [3]. However, this dataset introduces an innovative perspective by combining multiple data modalities, enabling a deeper analysis of collaborative dynamics within agile teams.

## 2 Background

Agile methodologies have transformed how teams collaborate in dynamic, iterative development environments. Frameworks such as Scrum [4], Kanban [5], Extreme Programming (XP) [6], and Lean [7] emphasize continuous communication, flexibility, and delivering incremental value. Team collaboration is essential in these settings, as decision-making occurs rapidly and adaptively in response to project needs. Practices such as daily stand-up meetings, iteration planning, and task estimation using techniques like planning poker foster active and coordinated participation among team members [8].

Since collaboration is a central pillar of agile methodologies, it is essential to have tools that allow for the objective measurement and analysis of interactions among team members. Although there are datasets that rec certain aspects of agile teams–such as task metrics, estimations, and completion times [3]—they do not comprehensively capture verbal and non-verbal interactions nor the participants’ subjective perceptions within a controlled environment. Multimodal Learning Analytics (MMLA) enables the study of collaborative processes by integrating data from diverse sources such as video, audio, digital activity logs, and physiological signals. Several datasets have been developed to study interaction and collaboration in learning environments. For instance, MUTLA provides synchronized learning records, including video and EEG signals to as- sess student engagement [9], and the Exploring Interactions and Regulations in Collaborative Learning dataset collects multimodal signals from high school students to analyse collaborative learning dynamics [10].

Another example of a relevant resource is the Math Data Corpus, which was introduced as part of the Second International Workshop on Multimodal Learning Analytics, held within the International Conference on Multimodal Interaction (ICMI 2013). This corpus collects multimodal recordings of students interacting while they solve mathematical problems. The recordings comprise speech, digital handwriting captured with electronic pens, and sequences of images. This collection allows for the analysis of communication and problem- solving patterns, as well as coding’s related to problem segmentation and the correctness of solution processes [11].

Despite the growing availability of datasets focused on education and collaboration in academic settings, no multimodal datasets specifically address collaboration within agile teams. In previous studies [12,13], we have explored the use of multimodal analytics to assess communication in agile practices such as planning poker; however, no publicly available datasets currently exist that enable a comprehensive analysis of these interactions for the scientific community.

## 3 Data Description

In this work, we present a new multimodal dataset focused on agile teams in face-to-face environments, aiming to capture data from multiple sources, including video, audio, automatic transcriptions, visual interactions, body posture, and signals of facial and hand synchrony. Data were collected from 19 groups of four people each, participating in two collaborative activities designed to assess team dynamics without explicit coordination and using planning poker, a consensus-based estimation technique used in agile software development. For each group, specific files were generated containing user tracking, facial, hand, and posture data, transcriptions, and visual attention directions, all in .parquet format. Additionally, the dataset includes audio recordings in .wav, processed result videos in .mp4, and a .zip archive with a comparative analysis between the transcription and user annotations (Table 1 provides a comprehensive summary of all dataset files and their specific purposes).

**Table 1:** Summary of dataset files and their purpose.

File	Format	Purpose	Method
*.userTracker.parquet	Parquet	Participant identification and tracking during the sessions.	YOLO and DeepSort were used for general recognition and tracking, with manual review for ID assignment per participant.
*.faceTracker.parquet	Parquet	Face tracking for facial expression analysis and presence detection.	MediaPipe Solutions was used, specifically the face_mesh model, to extract facial landmarks.
*.poseTracker.parquet	Parquet	Body posture tracking of each participant.	MediaPipe Solutions was used, specifically the pose model, to extract body keypoints.
*.handsTracker.parquet	Parquet	Hand detection and tracking for gesture analysis.	MediaPipe Solutions was used, specifically the hand model, to extract hand keypoints.
*.faceDirection.parquet	Parquet	Estimation of head orientation to infer gaze direction.	Calculated using camera angles, largely avoiding the use of the Rodrigues function for direction vector handling [14].
*.ObservedUser.parquet	Parquet	Data on where each participant directs their visual attention.	Procedure detailed in the associated study.
*.transcription.parquet	Parquet	Time-aligned automatic transcriptions with speaker diarization.	Transcriptions were generated from the .wav audio using WhisperX and manually reviewed.
mimicry_indicators.xlsx	XLSX	Group-level labeling of facial and bodily mimicry events.	Manual annotation of each action.
perception.xlsx	XLSX	Data on participants’ perceived equity during the activity.	Personal survey completed by each participant who agreed to respond.
results_transcription_vs_ observed_user.zip	ZIP	Comparison between spoken content (transcription) and observed gaze direction.	Counting based on specific predefined rules.
*.wav	WAV	Audio recording of the collaborative sessions.	Processed using moviepy.
*.mp4	MP4	Summary video showing activity results for each group.	CV2 was used to visualize tracking points and extract data from the corresponding files.

The dataset also includes manually labelled events of mimicry (facial and bodily) for the experimental groups (excluding control groups), stored in .xlsx format. In addition, perceived equity data reported by some participants are provided, also in .xlsx format. This dataset enables in-depth analysis of real- time collaborative dynamics, the evaluation of agile methodologies’ effectiveness, and the exploration of communication, leadership, and decision-making patterns—constituting a valuable resource for predictive modelling and the enhancement of team performance.

It is important to note that the control groups were not manually reviewed, as they were considered internal test cases. Participants in these groups had previously taken part in similar experiments, and therefore, their records are not considered fully independent.

The descriptive analysis of the dataset is structured in two complementary dimensions that capture fundamental aspects of collaborative interaction in agile teams: (1) visual attention metrics, which reveal gaze patterns among participants, and (2) behavioural synchrony metrics, which quantify bodily and facial mimicry. When analysed together, these dimensions allow for the characterization of both the quality and the temporal dynamics of collaboration from complementary perspectives, offering a comprehensive view of group interaction processes.^5^

### 3.1 Visual Attention Metrics

**Fig. 1:**
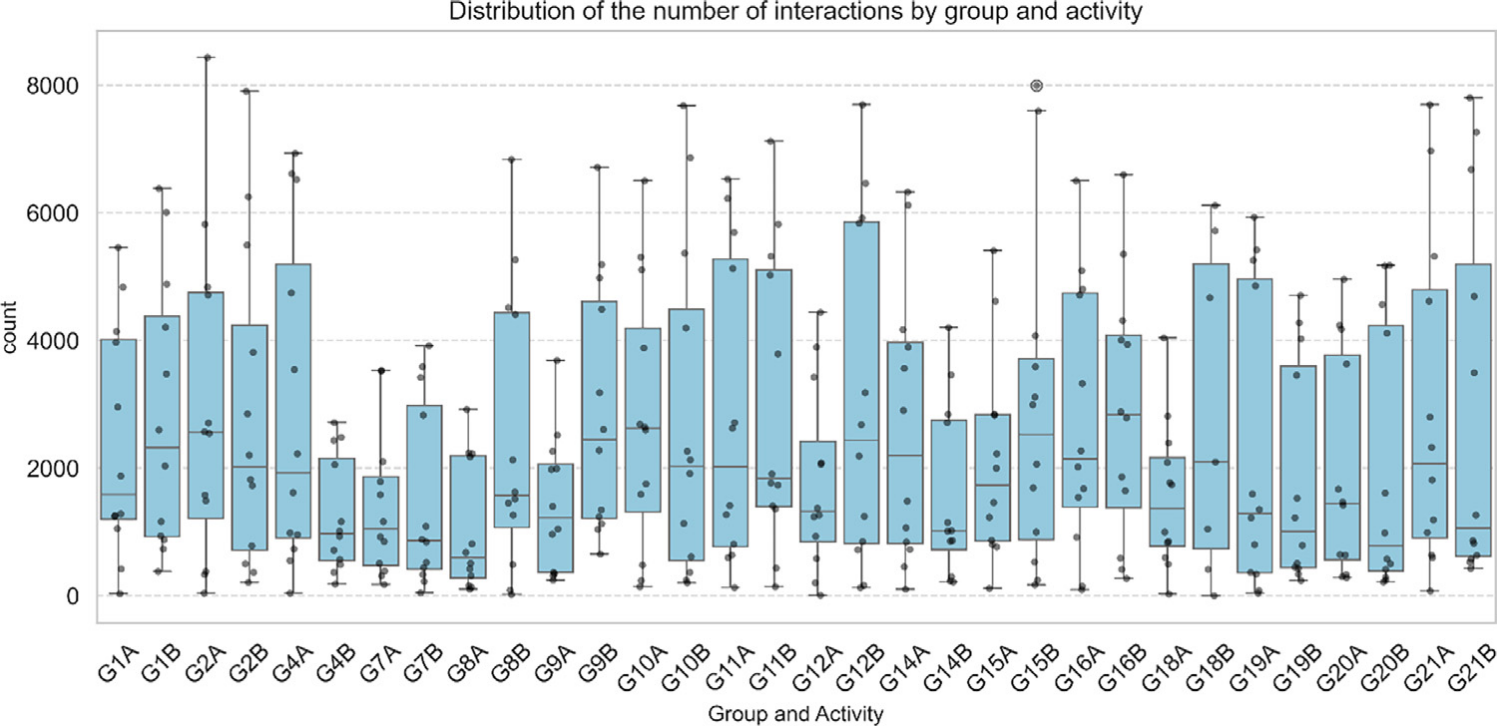
Distribution of the number of interactions by group and activity.

An analysis of the number of observations per group and activity was conducted. Significant variability was observed across groups, with outliers indicating sessions with an unusually high number of observations, as shown in Fig. 1. This heterogeneity in observation patterns suggests notable differences in the interaction dynamics among the analysed teams.

**Fig. 2:**
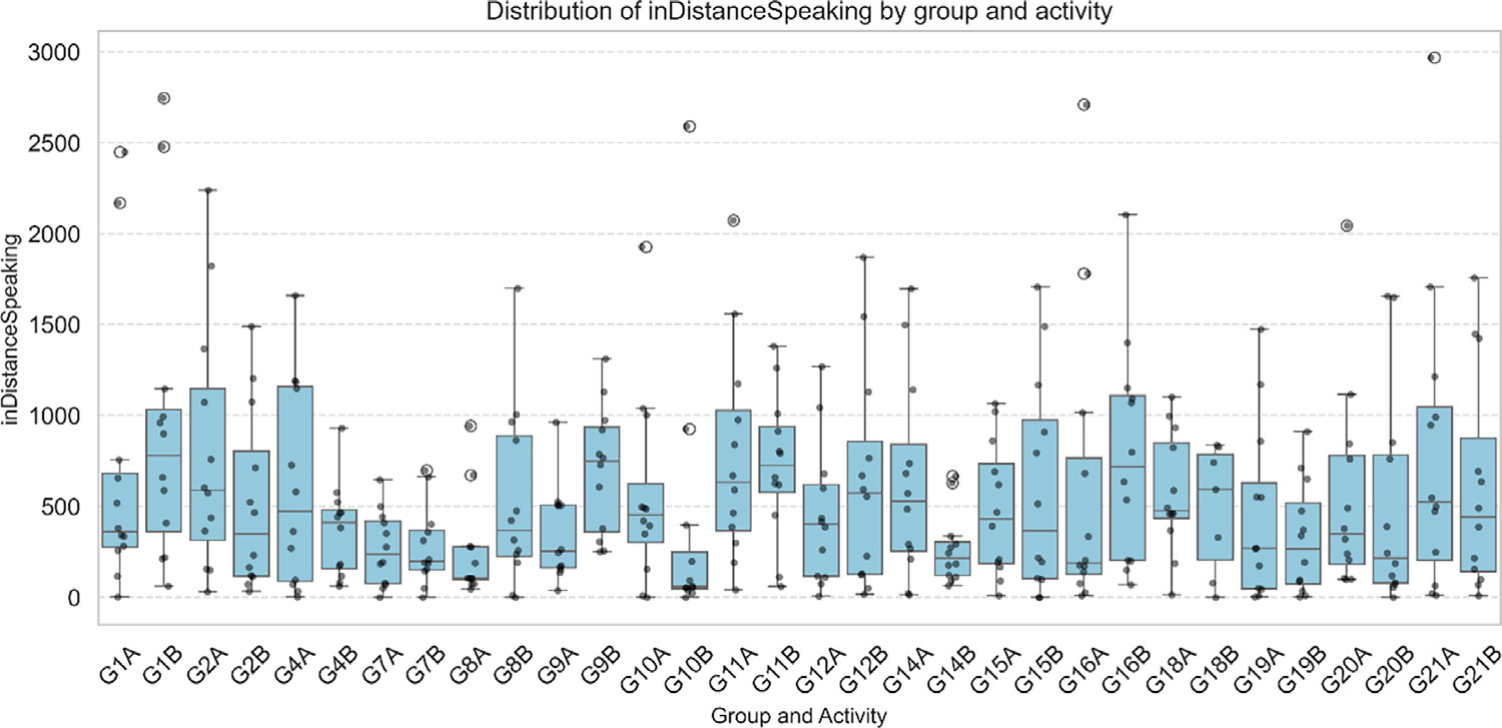
Distribution of inDistanceSpeaking by group and activity.

To further explore the nature of the previously described interactions, specific variables were analysed to characterize the quality of visual attention. The variables inDistance and inDistanceSpeaking reveal notable differences in visual proximity between participants. inDistance represents the number of frames in which one participant looks at another at a short facial distance. In contrast, inDistanceSpeaking measures how often this gaze occurs when the observed participant speaks, providing a more refined metric of focused attention during verbal communication (see Fig. 2).

**Fig. 3:**
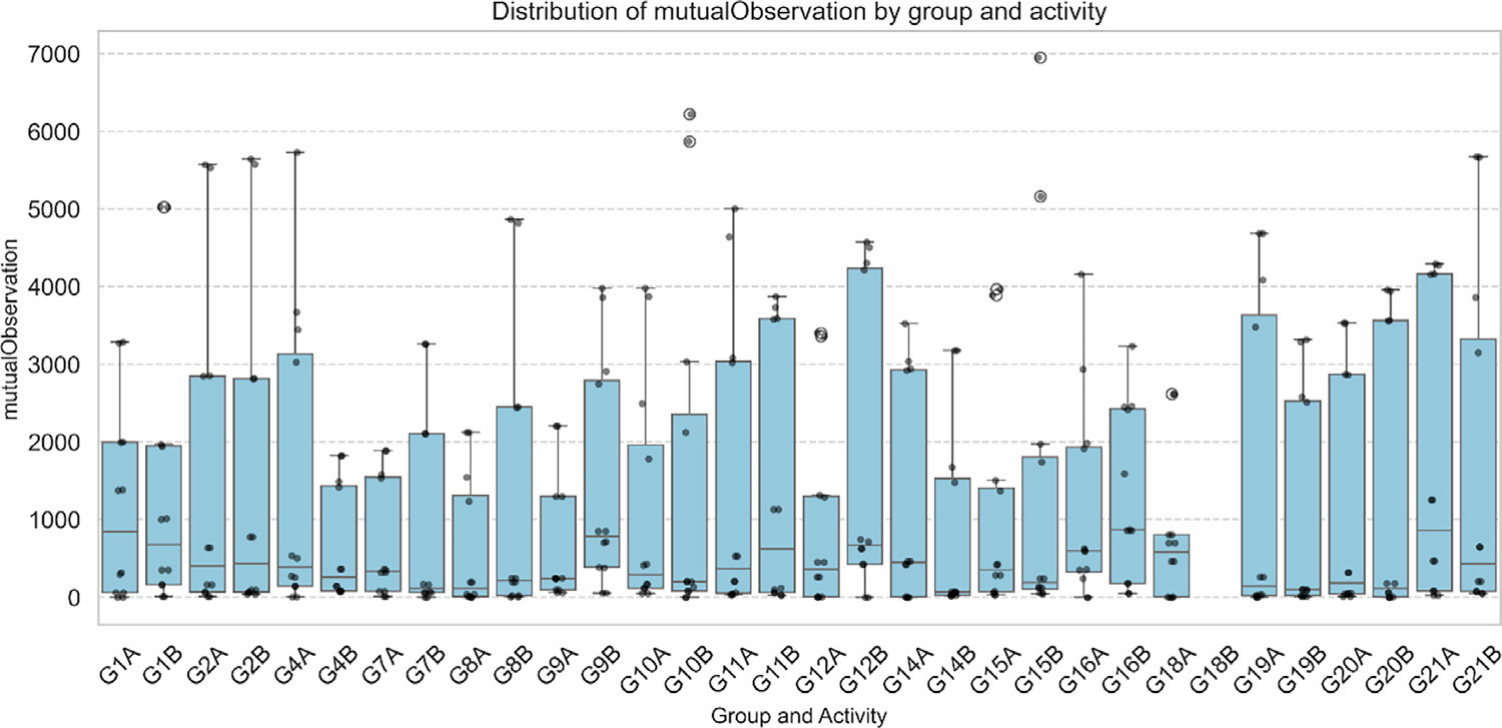
Distribution of mutualObservation by group and activity.

Complementarily, the variable isSpeaking captures the number of frames in which one user looks at another while the latter is speaking, which can be interpreted as an indicator of active listening in a collaborative context. On the other hand, the variable mutualObservation measures instances where two users look at each other simultaneously, representing moments of shared attention that may be associated with increased coordination or agreement. A noteworthy finding is that groups with higher mutualObservation values also tend to exhibit greater verbal interactions, suggesting a potential relationship between reciprocal visual attention and communicative engagement (Fig. 3).

Table 2 presents the descriptive statistics of these variables, highlighting significant variability across all indicators. This variability can be interpreted as reflecting the diverse interaction styles among the teams examined.

**Table 2:** Descriptive statistics of the analyzed variables.

Variable	Mean	Median	Standard Deviation	Minimum	Maximum
count	2349.31	1622.0	2086.26	1.0	8440.0
inDistance	1973.50	1363.0	1769.88	1.0	8433.0
inDistanceSpeaking	548.84	390.0	546.82	0.0	2969.0
isSpeaking	624.07	443.0	620.88	0.0	3354.0
mutualObservation	1216.52	318.0	1604.63	0.0	6953.0

**Fig. 4:**
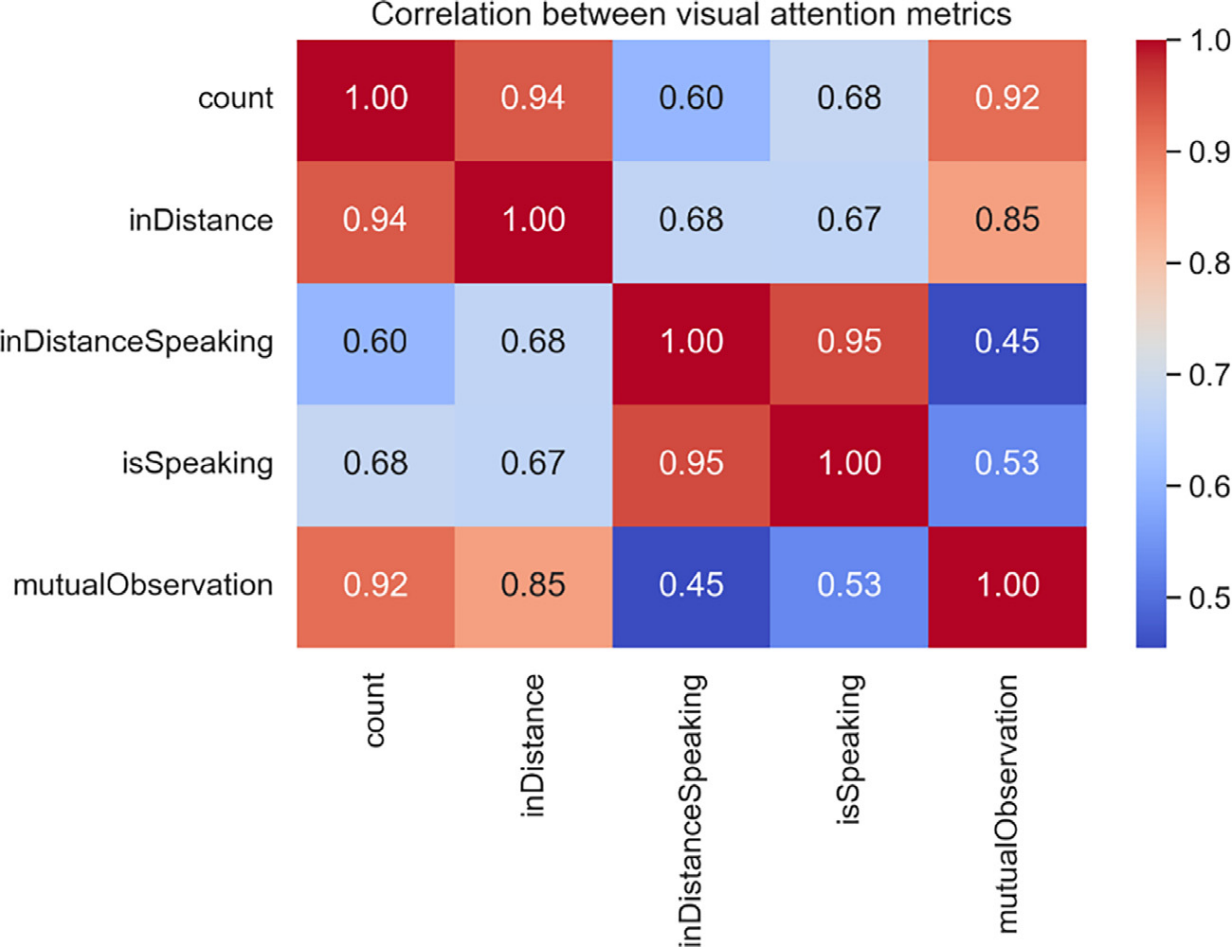
Correlation between visual attention metrics.

The correlation analysis between the metrics (Fig. 4) reveals patterns that may be relevant for understanding visual attention dynamics. A strong correlation was observed between count and inDistance (0.94), suggesting that the total number of interactions may be linked to visual proximity. Similarly, there is a robust correlation between inDistanceSpeaking and isSpeaking (0.95), which is expected given that both metrics capture aspects related to attention during speech. However, the more moderate correlation between mutualObservation and inDistanceSpeaking (0.45) is particularly interesting, as it may indicate that mutual observation and attention during speech reflect different facets of collaborative dynamics.

### 3.2 Behavioral Synchrony Metrics

While visual attention metrics provide valuable insights into gaze patterns among participants, they do not fully capture the bodily dimension of group synchrony. An analysis of bodily and facial mimicry was incorporated to obtain a more comprehensive view of collaborative dynamics. This complements the previous metrics by capturing shared behaviors between participants within short time intervals. The gender composition in the group identifiers (e.g., ”01A- MMMF”) indicates the gender distribution of participants, where M represents male and F represents female (for example, MMMF means three males and one female; MMFF means two males and two females).

These events were classified as synchronous (identical actions co-occurring) or asynchronous (imitation within a 6-second window). Table 3 presents the descriptive statistics for these mimicry events, allowing for a distinction between behaviours that occur at the same time and those that reflect a temporal influence between participants. It’s important to clarify that an “event” represents a distinct occurrence of mimicry behaviour, while “instances” count the specific manifestations of synchronous or asynchronous mimicry within that event. This explains why the total number of events (2560) doesn’t equal the sum of sync and async instances (3275), as a single event can contain multiple instances of both types. For example, if one participant makes a gesture and three others imitate it simultaneously, this would count as one event but three synchronous instances. This distinction is fundamental for understanding the nature of mimicry as an interactive phenomenon, beyond mere behavioural coincidence.

**Table 3:** Descriptive statistics of mimicry. Note: A single event may contain multiple instances of sync and async mimicry patterns.

Type of Mimicry	Events	Sync Instances	Async Instances
Body	1129	1322	241
Face	1431	1485	227
Total	2560	2807	468

Analysis of the complete dataset reveals a pronounced dominance of synchronous behaviours, with synchronous instances representing 86.24% of all mimicry events compared to 13.76% asynchronous instances. This pattern remains remarkably consistent across behaviour types, with body mimicry showing 85.3% synchronous coordination and face mimicry demonstrating 87.1% synchronous events. The overwhelming prevalence of synchronous behaviours indicates a strong natural propensity toward immediate behavioural coordination rather than delayed imitation, suggesting that collaborative teams inherently gravitate toward simultaneous coordination mechanisms during agile interactions.

Beyond these general patterns, the analysis reveals significant variations in synchronization rates based on group gender composition. Groups with different gender distributions demonstrate markedly different coordination levels, with female-majority groups achieving substantially higher synchronization rates compared to male-majority groups. Table 4 presents synchronization rates across different gender compositions, showing that groups with majority female participation reach 94.66% synchronization compared to 85.23% for male-majority groups—a difference of 9.43 percentage points. Mixed-gender groups with balanced representation show intermediate synchronization levels at 89.14%, suggesting that gender diversity influences collaborative dynamics through enhanced behavioural coordination mechanisms.

**Table 4:** Synchronization rates by group gender composition.

Composition	Gender Ratio	Sync %	Groups	Total Events
MFFF	1M, 3F	94.7	2	131
FMMF	2M, 2F	92.8	2	207
MFFM	2M, 2F	92.2	2	103
MMFM	3M, 1F	86.8	4	561
MMMF	3M, 1F	86.7	2	286
MMFF	2M, 2F	85.1	2	261
MMMM	4M, 0F	84.5	18	1,889
By Majority Type				
Female Majority		94.7	2	131
Balanced		89.1	6	571
Male Majority		85.2	24	2,736

The progression from male-dominated groups (MMMM: 84.5%) to female-majority groups (MFFF: 94.7%) represents a 10.2 percentage point increase in synchronization, indicating that gender composition constitutes a meaningful factor in team coordination dynamics. This effect appears to be gradual rather than binary, with synchronization rates generally increasing as the proportion of female participants increases, supporting hypotheses about differential collaborative behaviours and social coordination mechanisms across gender compositions.

Table 5 presents a detailed summary of mimicry events by group, distinguishing by type (bodily or facial) and temporal nature (synchronous or asynchronous). This granularity level allows for identifying specific synchronization patterns within each team.

**Table 5:** Mimicry events by group and type, differentiated by synchrony.

Group	Type of Mimicry	Events	Sync	Async
01A-MMMF	Body	52	46	14
01A-MMMF	Face	28	27	10
01B-MMMF	Body	18	25	3
01B-MMMF	Face	152	150	11
02A-MMMM	Body	12	8	8
02A-MMMM	Face	124	124	2
02B-MMMM	Body	54	54	4
02B-MMMM	Face	109	111	5
04A-MMMM	Body	2	2	0
04A-MMMM	Face	101	119	11
04B-MMMM	Body	21	25	2
04B-MMMM	Face	5	3	4
07A-MFFF	Body	2	1	1
07A-MFFF	Face	6	5	2
07B-MFFF	Body	69	96	4
07B-MFFF	Face	22	22	0
08A-MMMM	Body	14	6	13
08A-MMMM	Face	3	2	2
08B-MMMM	Body	18	34	6
08B-MMMM	Face	73	98	12
09A-MMMM	Body	0	0	0
09A-MMMM	Face	16	16	2
09B-MMMM	Body	23	17	15
09B-MMMM	Face	48	44	7
10A-MMMM	Body	22	21	8
10A-MMMM	Face	50	47	10
10B-MMMM	Body	46	56	10
10B-MMMM	Face	79	82	13
11A-MMMM	Body	85	120	29
11A-MMMM	Face	36	33	8
11B-MMMM	Body	16	17	9
11B-MMMM	Face	91	91	21
12A-MMFF	Body	82	92	12
12A-MMFF	Face	25	21	8
12B-MMFF	Body	20	25	9
12B-MMFF	Face	26	23	9
14A-MMFM	Body	32	31	11
14A-MMFM	Face	60	72	14
14B-MMFM	Body	4	7	1
14B-MMFM	Face	62	66	14
15A-MMMM	Body	67	95	20
15A-MMMM	Face	36	34	10
15B-MMMM	Body	133	148	17
15B-MMMM	Face	45	43	17
16A-MMFM	Body	148	174	7
16A-MMFM	Face	25	25	3
16B-MMFM	Body	36	40	9
16B-MMFM	Face	68	72	14
18A-MMMM	Body	12	6	8
18A-MMMM	Face	0	0	0
18B-MMMM	Body	3	3	0
18B-MMMM	Face	10	10	0
19A-FMMF	Body	7	6	2
19A-FMMF	Face	40	54	7
19B-FMMF	Body	9	33	4
19B-FMMF	Face	27	26	1
20A-MFFM	Body	20	18	2
20A-MFFM	Face	5	5	1
20B-MFFM	Body	39	47	4
20B-MFFM	Face	14	14	1
21A-MMMM	Body	28	26	3
21A-MMMM	Face	11	13	2
21B-MMMM	Body	35	43	6
21B-MMMM	Face	34	33	6

### 3.3 Complementarity of Multimodal Data

Visual attention metrics (such as inDistance, inDistanceSpeaking, isSpeaking, and mutualObservation) capture quantifiable patterns regarding the direction, duration, and intensity of attention among participants. These data are essential for understanding how attention is distributed during collaborative sessions and can be correlated with performance variables or team roles.

On the other hand, mimicry metrics (bodily and facial, synchronous and asynchronous) provide a window into the behavioural synchronization processes that occur during collaboration. These data complement visual metrics by adding a bodily dimension to the characterization of interaction, allowing researchers to explore hypotheses about interpersonal synchronization and its relationship with team effectiveness.

This multimodal approach extends traditional datasets by incorporating not only what is said (through transcriptions) or observed (through gaze patterns), but also how teams synchronize bodily. The availability of these different layers of information opens up new possibilities for studying group dynamics in agile methodologies, making it a valuable resource for researchers interested in modelling and predicting collaborative behaviour in software development environments.

## 4 Experimental Design, Materials and Methods

The experiments aimed to investigate the research question: How do different coordination techniques influence collaboration dynamics in agile development teams during user story estimation? [12]. This study employed a quantitative and exploratory approach, with a within-subjects design, were each subject was a team. Each team was subjected to two treatments: ad hoc estimation (without predefined coordination technique) and estimation using Planning Poker, which provides an structured coordination.

**Fig. 5:**
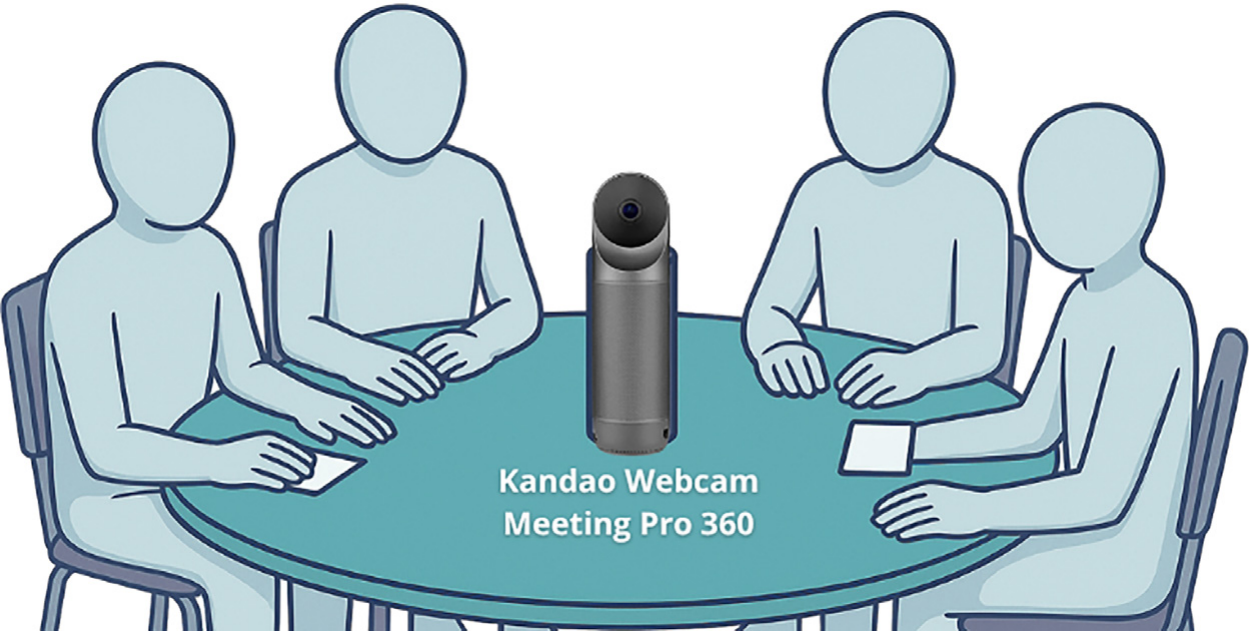
Example of a group activity viewed from the outside.

The experiments were conducted between August and November 2023 in a controlled laboratory at the Universidad de Valparaíso. The experimental procedure began with a public recruitment process targeting computer engineering students with prior knowledge of agile methodologies. However, two teams were excluded from the analysis for not adhering to the experimental guidelines, resulting in 19 usable datasets. After a training phase covering the techniques to be used, each team participated in two collaborative activities involving both treatments. The physical setup placed participants around a circular table to facilitate audiovisual recording using a Kandao Meeting Pro 360° camera and an omnidirectional microphone, as shown in Fig. 5. During each session, which lasted up to 10 minutes, the teams were tasked with reaching a consensus on the complexity of a user story.

**Fig. 6:**
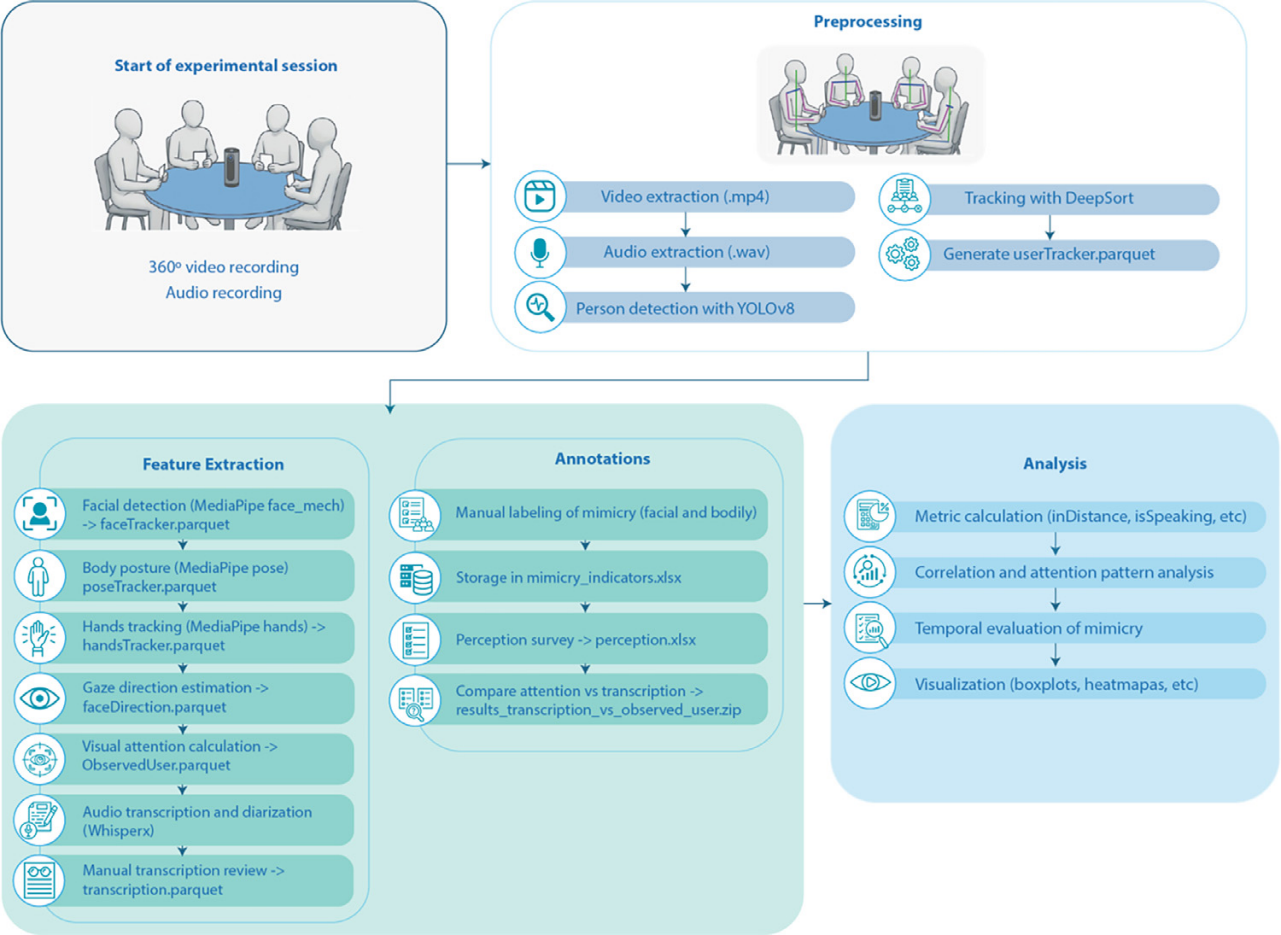
Data processing workflow for multimodal analysis of agile team interactions. The diagram illustrates the complete pipeline from initial experimental session recording through preprocessing, parallel feature extraction and manual annotation processes, to final analysis of collaborative behaviours.

Fig. 6 illustrates the comprehensive data processing workflow employed in this study. The process begins with the experimental session, where 360° video and audio recordings are captured simultaneously. During preprocessing, the raw media is extracted into appropriate formats (mp4 for video, wav for audio), followed by person detection using YOLOv8 and tracking with DeepSort to generate participant tracking data. The workflow then branches into two parallel streams: Feature Extraction and Annotations. The Feature Extraction path includes facial detection, body posture tracking, and hand movement analysis using MediaPipe, along with gaze direction estimation, visual attention calculation, and audio transcription with WhisperX. Important manual reviews by junior and senior judges are conducted at key points in the workflow: in the Feature Extraction branch to verify correct participant tracking identification, and to review transcription accuracy. Meanwhile, the Annotations branch involves manual labelling of mimicry behaviours, perception surveys, and comparative analysis between attention patterns and transcribed speech. Finally, the Analysis phase encompasses metric calculation, correlation analysis, temporal evaluation of mimicry patterns, and data visualization through various graphical representations. This methodical approach ensures comprehensive multimodal data collection and systematic analysis of collaborative interactions.

During the experimental sessions, we controlled for additional factors, including the order in which user stories were presented for estimation to reduce potential learning effects. We kept variables such as team roles, task difficulty, and the physical environment consistent across all groups. The dependent variables measured included total speaking time, individual speaking time for each participant, and attention distribution, which were analysed to identify patterns of group-level focus.

Data collection involved the video and audio recording of sessions for subsequent analysis using multimodal analytics tools. Participant detection was performed using YOLOv8, while tracking was carried out with DeepSort. MediaPipe was used to extract facial landmarks and estimate head orientation, which was then used to compute gaze direction vectors in 3D space based on data from a panoramic camera. For audio analysis, we employed WhisperX to generate accurate transcriptions and perform speaker diarization with word- level temporal alignment. These transcriptions were stored in Parquet format and manually reviewed to correct any assignment and segmentation errors. The results were validated through an iterative review process, pilot studies, and mathematical verification with synthetic data, ensuring the reliability of the collected measurements. The dataset supporting these findings is publicly available in Mendeley Data [17,18].

## Limitations

• The sample is limited to university students, which may not fully capture the dynamics of professional teams. However, previous research has shown that, when carefully designed, experiments involving students can produce results comparable to those obtained with professionals—especially when the development approach is new to both groups [15,16]. Thus, using students remains a valid and accepted simplification in empirical software engineering research.
• The controlled experimental setting may not fully reflect real-world working conditions, which could limit ecological validity.

## Ethics Statement

This study was approved by the Institutional Ethics Committee for Re- search Involving Human Subjects at the Universidad de Valparaíso (protocol code CEC-UV 236-21). All participants signed an informed consent form before participating in the study, ensuring they understood and agreed to the terms. No financial compensation was offered, emphasizing the voluntary nature of participation and the students’ interest in contributing to research on software engineering and teamwork dynamics. The study was conducted by the principles of the Declaration of Helsinki, ensuring compliance with ethical standards for research involving human subjects. Furthermore, data were anonymized, and privacy was safeguarded throughout the processing and analysis stages.

## Declaration of Generative Ai and Ai-Assisted Technologies in the Writing Proces

During the preparation of this work, the authors used Grammarly Editor to improve readability and language. After using this tool, the authors reviewed and edited the content as needed and took full responsibility for the publication’s content.

## CRediT authorship contribution statement

**Diego Miranda:** Writing – review & editing, Writing – original draft, Software, Validation, Formal analysis, Visualization. **Carlos Escobedo:** Validation, Data curation, Writing – original draft, Writing – review & editing. **Dayana Palma:** Validation, Data curation, Writing – original draft. **Rene Noel:** Writing – review & editing, Supervision, Methodology, Formal analysis, Conceptualization, Project administration. **Adrián Fernández:** Validation, Data curation, Writing – original draft. **Cristian Cechinel:** Writing – review & editing, Methodology. **Jaime Godoy:** Data curation. **Roberto Munoz:** Writing – review & editing, Formal analysis, Validation, Supervision, Methodology, Conceptualization, Project administration, Funding acquisition.

## Footnotes

1 WhisperX: https://github.com/m-bain/whisperX.

2 MediaPipe: https://developers.google.com/mediapipe.

3 YOLOv8: https://github.com/ultralytics/yolov8.

4 DeepSort: https://pypi.org/project/deep-sort-realtime/.

## Footnotes

5 moviepy: https://pypi.org/project/moviepy/.
